# Polypropylene Biocomposites with Boron Nitride and Nanohydroxyapatite Reinforcements

**DOI:** 10.3390/ma8030992

**Published:** 2015-03-10

**Authors:** Kai Wang Chan, Hoi Man Wong, Kelvin Wai Kwok Yeung, Sie Chin Tjong

**Affiliations:** 1 Department of Physics and Materials Science, City University of Hong Kong, Tat Chee Avenue, Kowloon, Hong Kong; E-Mail: kaiwchan8-c@my.cityu.edu.hk; 2 Department of Orthopedics and Traumatology, Li Ka Shing Faculty of Medicine, the University of Hong Kong, Hong Kong; E-Mails: kwhoiman@gmail.com (H.M.W.); wkkyeung@hku.hk (K.W.K.Y.); 3 Shenzhen Key Laboratory for Innovative Technology in Orthopedic Trauma, the University of Hong Kong Shenzhen Hospital, 1 Haiyuan 1st Road, Shenzhen 518053, China

**Keywords:** boron nitride, hydroxyapatite, nanocomposite, osteoblast, cytotoxicity, hybridization

## Abstract

In this study, we develop binary polypropylene (PP) composites with hexagonal boron nitride (hBN) nanoplatelets and ternary hybrids reinforced with hBN and nanohydroxyapatite (nHA). Filler hybridization is a sound approach to make novel nanocomposites with useful biological and mechanical properties. Tensile test, osteoblastic cell culture and dimethyl thiazolyl diphenyl tetrazolium (MTT) assay were employed to investigate the mechanical performance, bioactivity and biocompatibility of binary PP/hBN and ternary PP/hBN-nHA composites. The purpose is to prepare biocomposite nanomaterials with good mechanical properties and biocompatibility for replacing conventional polymer composites reinforced with large hydroxyapatite microparticles at a high loading of 40 vol%. Tensile test reveals that the elastic modulus of PP composites increases, while tensile elongation decreases with increasing hBN content. Hybridization of hBN with nHA further enhances elastic modulus of PP. The cell culture and MTT assay show that osteoblastic cells attach and proliferate on binary PP/hBN and ternary PP/hBN-20%nHA nanocomposites.

## 1. Introduction

In recent years, the global demand for artificial human bone replacements has been ever increasing due to a surge in the number of patients suffering from aging, bone disease and injury. Most orthopedic implants are made of metallic materials, including austenitic 316L stainless steel, cobalt-chromium and titanium-based alloys, due to their high mechanical strength and good ductility. However, the Young’s modulus of such metallic alloys far exceeds that of human bones. This creates a stress shielding effect of the surrounding bone tissue, causing the implant to carry a higher proportion of the applied load. Consequently, bone resorption and loosening of the metallic implant can result in the failure of the replacement. Moreover, human body fluids with about 0.9 wt% sodium chloride at 37 °C are hostile to metallic alloys. Thus, metallic implants may undergo electrochemical dissolution or corrosion upon exposure to human body fluids, releasing metallic ions that induce inflammatory response, allergy and cytotoxicity.

In general, nickel ion is the main cause of allergy, followed by cobalt and chromium [1,2]. Furthermore, Cr^3+^ and Co^2+^ ions can bind to several cellular proteins, induce oxidation and impair their biological function, thereby causing cell death and tissue damage [3]. Generally, stainless steels exhibit good wear and corrosion resistance in aqueous environments due to the formation of thin passive oxide/hydroxide films on their surfaces [4,5,6]. Unfortunately, chloride ions can breakdown the passive films of stainless steels, causing pitting and crevice corrosion and creating anodic dissolution in localized regions of steels. Cobalt-chromium and 316L steel are susceptible to localized corrosion in physiological saline Ringer’s solution. Ti-based alloy, such as Ti-6Al-4V, is more corrosion resistant, but inferior wear behavior is its main disadvantage [7]. These potential risks and health hazards with metallic devices have motivated materials scientists to search for other materials with good biocompatibility and no cytotoxicity.

Polymers usually find useful applications in biomedical sectors, due to their being lightweight, the ease of fabrication and the relatively low cost [8,9,10,11]. Their tensile stress and modulus can be monitored by adding fillers of micrometer sizes [12,13,14,15,16,17,18]. Thus, the composite approach is an effective route for producing polymer biomaterials with desired mechanical properties for bone replacements. As an example, Bonfield and coworkers added 40 vol% hydroxyapatite microfillers to high-density polyethylene to form HAPEX^TM^ composite [19,20]. Hydroxyapatite resembles the mineral component of human bones and is responsible for their mechanical strength and stiffness. However, synthetic hydroxyapatite microparticles (mHA) usually debond from the polymer matrix during the tensile test [21]. Moreover, large mHA particles usually break into small fragments upon tensile loading. These effects directly cause ineffective load transfer from the matrix to mHA fillers, resulting in low tensile strength and failure of the composite [21].

Recent advances in nanoscience and nanotechnology have led to the development and creation of functional nanomaterials with unique chemical, physical mechanical and biological characteristics. In the past decade, the use of nanomaterials in healthcare and biomedical sectors has been rapidly growing due to their potential applications in antimicrobial, bioimaging, drug delivery and orthopedic sectors [22,23,24]. As recognized, bone tissues are composed of nano-hydroxyapatite (nHA) platelets and collagen fibers. Accordingly, synthetic nanohydroxyapatite particles have been used as reinforcing fillers for non- and degradable polymers to form nanocomposites for biomedical applications, e.g., bone plates and bone scaffolds [25,26,27,28,29,30,31]. The attachment and growth of osteoblasts are significantly enhanced on the surface of nHA fillers. In addition, the filler loadings in thermoplastic polymers can be drastically reduced by adding nHA particles. The incorporation of 20 wt% nHA (6.67 vol%) to polypropylene (PP) gives rise to good biocompatibility [32]. The nHA/polymer nanocomposites exhibit better mechanical properties over conventional mHA/polymer composites at the same filler loading level.

In previous studies, we simultaneously added nHA and carbon nanotubes (CNTs) or carbon nanofibers (CNFs) to PP to form biocomposites for bone replacements [32,33]. The addition of low CNT/CNF loadings to nHA/PP composites further enhances their mechanical performance due to the large aspect ratio and remarkable high stiffness of carbonaceous nanofillers. Generally, CNTs are compatible with biological cells, provided that they are firmly embedded within the matrix of polymer composites. However, standalone or individual CNT suspension is reported to be particularly toxic to biological cells, and the cytotoxicity increases with increasing nanotube doses [34,35,36]. This is because needle-like CNTs can penetrate through the cell membrane and finally reside in the nucleus. BN sheets can also be rolled up into nanotubes with cellular seeding and growth behaviors similar to those of CNTs [37]. In certain cases, boron nitride nanotubes are even more cytotoxic than CNTs [38,39]. Hexagonal boron nitride (hBN) with a layered structure like graphite generally exhibits excellent lubricant behavior, superior thermal and chemical stability and good biocompatibility [40,41]. hBN and titania have been used as a filler for chitosan acting as a protective coating for stainless steel substrate [42]. In this study, we attempted to use planar hBN and nanohydroxyapatite to reinforce PP to form biocomposites for bone replacements. Boron nitride with platelet morphology was selected in order to avoid the cytotoxicity of tubular boron nitride. The main interest in employing layered hBN in polymer hybrid composites was their high chemical stability, good processability and good biological activity [43]. No information is available in the literature on the biocompatibility and tensile behavior of the hBN platelet/PP nanocomposites and hBN-nHA/PP hybrids for biomedical applications. To the best of our knowledge, the present work is the first investigation of the development of PP nanocomposites reinforced with hBN platelets and nHA rods and their biocompatibility.

## 2. Experimental Section

### 2.1. Materials

**Figure 1:**
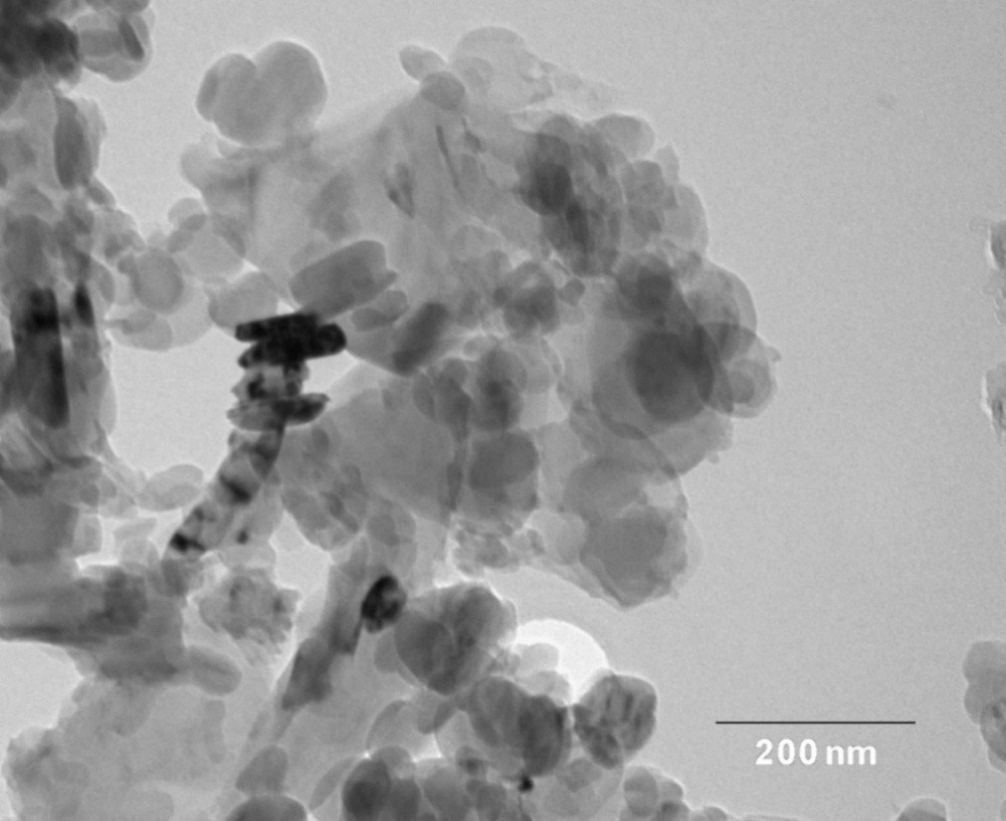
TEM micrographs of hexagonal boron nitride (hBN) showing platelet feature with sizes ranging from about 30 to 150 nm.

**Figure 2:**
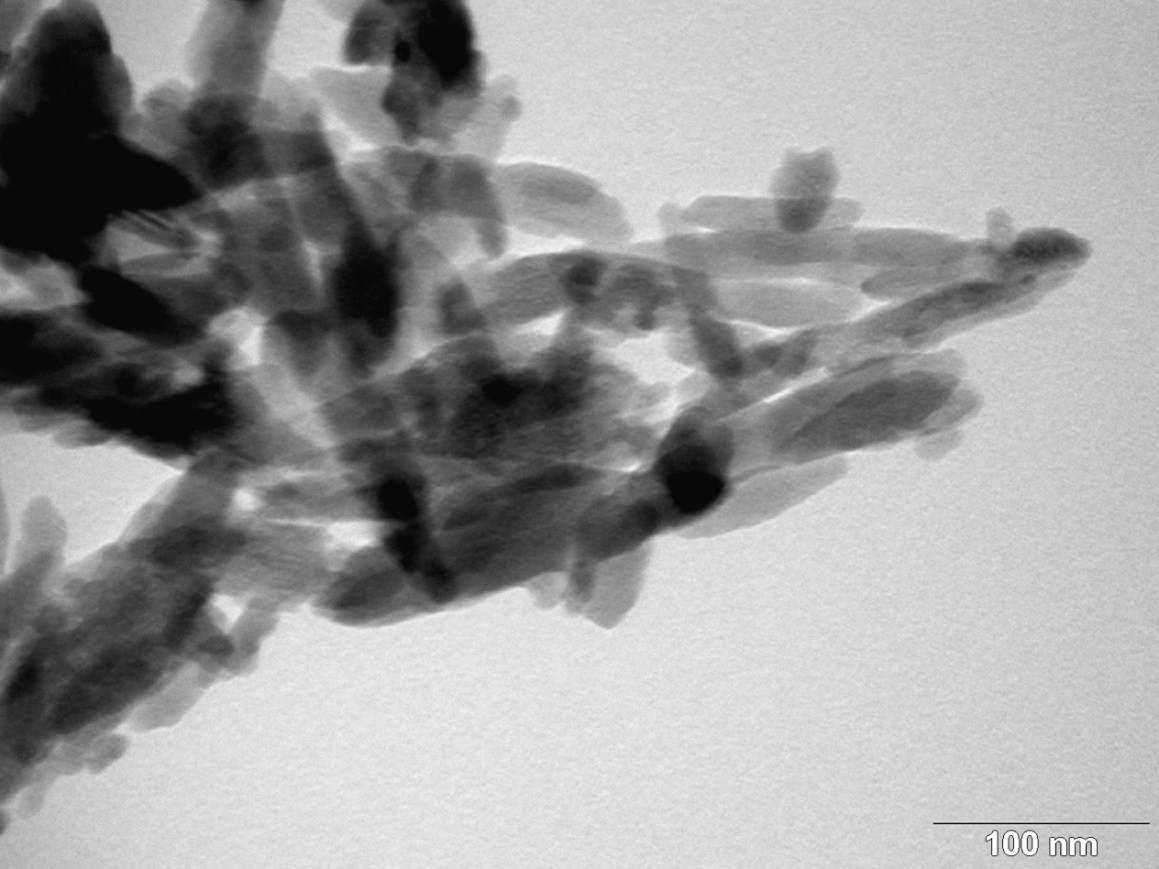
TEM micrograph of nHA showing rod-like feature with an average length of 90 nm.

Nanostructured & Amorphous Materials Inc. (Houston, TX, USA) supplied hBN powders for this study. Figure 1 shows the TEM image of hBN powders. TEM examination was performed using a Philips CM-20 TEM microscope (Philips, Amsterdam, The Netherlands) attached to an energy dispersive X-ray spectrometer. Apparently, hBN exhibited a platelet feature with sizes ranging from about 30 to 150 nm. Nanohydroxyapatite powders with a rod-like feature, *i.e*., a length of about 100 nm and a width of 20 nm, were purchased from Nanjing Emperor Nano Materials (Nanjing, China). Figure 2 shows the TEM image of nHA with rod-like feature. Polypropylene pellets for injection molding purpose (Mophlen HP 500N) were obtained from Basell (Jubail, Saudi Arabia). In general, surface coupling agents can enhance interfacial bonding between the filler and the matrix; however, they may induce cytotoxicity [44]. Both hBN and nHA fillers were not treated with organic surface coupling agents in order to avoid cytotoxicity of the biocomposites.

### 2.2. Preparation of Nanocomposites

Extrusion and injection molding are versatile and effective processes for large-scale manufacturing of PP-based composites [13,15,45]. Table 1 summarizes typical compositions of binary PP/hBN and ternary PP/hBN-nHA composites. Prior to melt-mixing, PP pellets were dried in an oven at 60 °C for 24 h. Melt compounding was first performed by feeding the material mixtures into a Brabender twin-screw extruder at a screw rotation speed of 30 rpm. The six barrel zones of this extruder were heated to temperatures ranging from 190 to 230 °C. Extruded products were sliced into small pellets and loaded into the Brabender extruder again. The purpose was to obtain effective mixing and homogeneous dispersion of reinforcing fillers in the polymer matrix. The products were pelletized again, dried overnight in an oven and subsequently molded into dog-bone tensile bars and circular disks using an injection molder (Toyo TI-50H, Akashi, Japan). The disks were primarily used for cell seeding and proliferation measurements. The composites were twice extruded for filler homogenization followed by injection molding; the matrix material could be degraded slightly. However, applied stress was mainly carried by the fillers of composites, thus minute matrix degradation would not affect overall mechanical performance of the composites greatly. Furthermore, nHA fillers were found to be very effective at improving the dimensional and thermal stability of PP [32]. Such a “three-step processing” strategy was also adopted by other researchers for making composite materials with improved physical and mechanical properties [46].

**Table 1:** The compositions of the polypropylene **(**PP) composites studied. nHA, nanohydroxyapatite.

Specimen	PP (wt%)	hBN (wt%)	nHA (wt%)
PP/5% hBN	95	5	0
PP/10% hBN	90	10	0
PP/15% hBN	85	15	0
PP /20% hBN	80	20	0
PP/20% nHA	80	0	20
PP/5% hBN-20% nHA	75	5	20
PP/10% hBN-20% nHA	70	10	20
PP/15% hBN-20% nHA	65	15	20

### 2.3. Material Characterization

Scanning electron microscopy (SEM) was employed to examine the morphologies of fillers, composites and osteoblasts. Both field-emission SEM (Jeol JSM-6335F, Tokyo, Japan) and conventional SEM (Jeol JSM 820, Tokyo, Japan) were used for this purpose. The surfaces of composite samples were deposited with a thin carbon film. Tensile experiments were performed at room temperature using an Instron tester (Model 5567, Norwood, MA, USA) at a crosshead speed of 10 mm·min^−1^ in accordance with ASTM D638-08 [47]. Young’s modulus of the samples was determined from the linear region of stress-strain curves. Five samples of each composition were used for testing, and the average values were evaluated.

### 2.4. Cell Seeding and Proliferation

Human osteoblasts (Saos-2) were seeded in Dulbecco’s Modified Eagle’s Medium (DMEM) supplemented with 10% fetal bovine serum, penicillin and streptomycin. The samples (4 × 4 × 1 mm) for cell cultivation and proliferation tests were sliced from injection molded disks into small rectangles. They were rinsed with 70% ethanol and phosphate-buffered saline (PBS) solutions. A suspension of Saos-2 containing 10^4^ cells was seeded on these samples placed in a 96-well plate and then kept in a humidified incubator with 5% CO_2_ in air at 37 °C for 4 and 7 days, respectively. The culture medium was refreshed every two days. Following the incubation, the samples were washed with PBS and fixed with 10% formaldehyde, followed by dehydration through a series of graded ethanol solutions and critical point drying. Once dry, they were deposited with a thin gold film and placed inside SEM.

The proliferation of osteoblasts on all specimens was assessed using the 3-(4,5-dimethylthiazol-2-yl)-2,5-diphenyltetrazolium bromide (MTT) assay in 96-well plates. A cell suspension with 10^4^ cells was introduced to cultured plates with and without samples followed by incubation in a humidified atmosphere of 5% carbon dioxide in air at 37 °C for 4 and 7 days, respectively. The culture medium was refreshed every 2 days. At selected cultivation periods, the medium was aspirated, then 10 μL of MTT solution (5 mg MTT:1 mL DMEM) was added to each well and incubated for 4 h for at 37 °C. At this stage, the tetrazolium ring of MTT salt was cleaved by the succinic dehydrogenase in mitochondria of viable osteoblasts, forming insoluble formazan crystals. The formazan was lastly dissolved in 10% sodium dodecyl sulfate (SDS)/0.01 M hydrochloric acid (100 µL). The absorbance or optical density (OD) of dissolved formazan was quantified spectrophotometrically at a wavelength of 570 nm using a multimode detector (Beckman Coulter DTX 880, Fullerton, CA, USA), with a reference wavelength of 640 nm. Wells with culture medium, MTT, SDS and osteoblasts were used as the control, while wells without osteoblastic cells were employed as a blank background. The samples were used for each test, and the results were expressed in terms of mean ± standard deviation (SD). MTT tests were repeated at least twice.

## 3. Results and Discussion

### 3.1. Morphology and Mechanical Behavior

**Figure 3:**
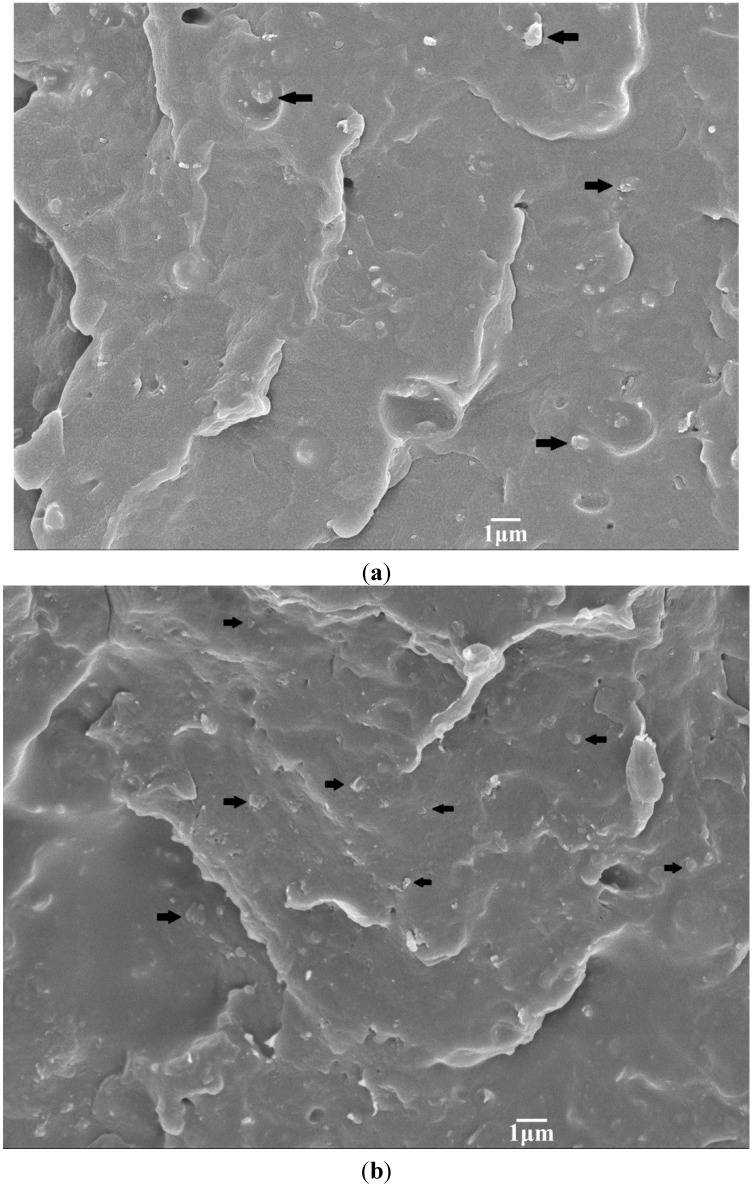
SEM micrographs showing fractured surfaces of (**a**) PP/5% hBN and (**b**) PP/15% hBN composites with a uniform dispersion of hBN fillers. Black arrow: hBN.

**Figure 4:**
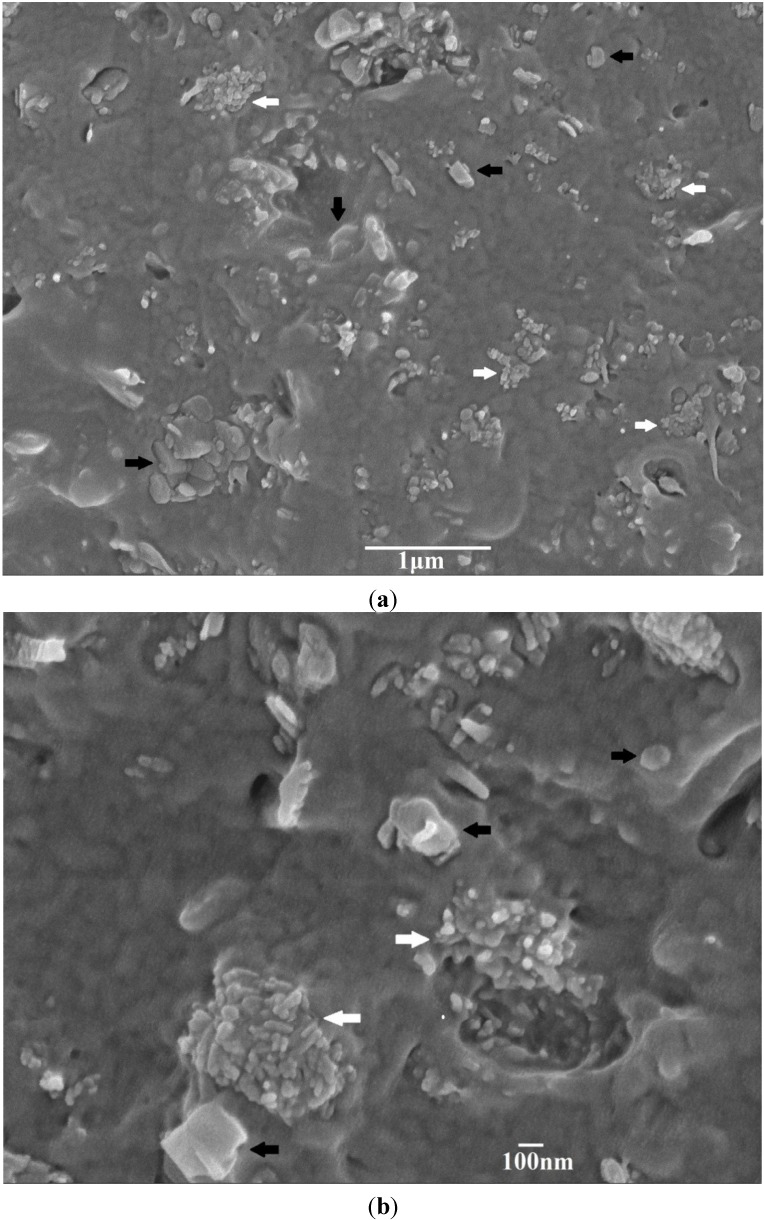
SEM micrographs showing fractured surface of PP/15% hBN-20% nHA hybrid at (**a**) low and (**b**) high magnifications. nHA aggregates can be readily seen. Black arrow: hBN; white arrow: nHA.

Figure 3a,b shows representative SEM images of binary PP/5% hBN and PP/15%hBN composites. Apparently, hBN fillers are dispersed uniformly in the PP matrix of these composites. The morphology of the typical PP/15% hBN-20%nHA hybrid at low and high magnifications is shown in Figure 4a,b, respectively. Similarly, hBN fillers are distributed homogeneously in the polymer matrix. However, aggregates of nHA fillers can be observed in the PP/15% hBN-20%nHA hybrid, due to its high nHA content, *i.e*., 20 wt%. Large nHA content is added to the PP hybrid in order to promote the adhesion and proliferation of osteoblasts. PP homopolymer is bioinert and, thus, ineffective for anchoring osteoblasts on its surface. As mentioned before, bone tissues are composed of nHA platelets and collagen fibers. Homogeneous dispersion of nHA in the PP matrix can be achieved by adding low nHA contents. However, such PP nanocomposites are unsuitable for biomedical implant applications due to their low biocompatibility. From our previous study, a minimum nHA content of 20 wt% is required for achieving good bioactivity and biocompatibility of the PP composites [32].

**Figure 5:**
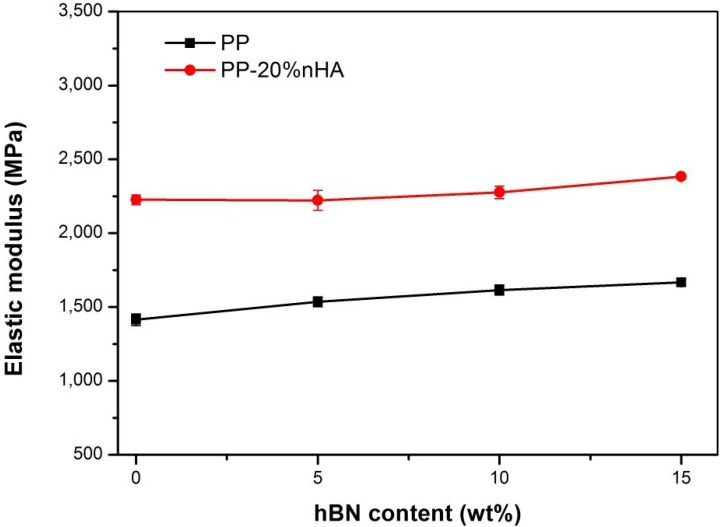
Elastic modulus *vs*. hBN content for PP/hBN and PP/hBN-nHA composites showing the stiffening effect of hBN.

The tensile test results for all specimens studied are tabulated in Table 2. For comparison, the tensile properties of high-density polyethylene (HDPE) composites reinforced with 10, 20, 30 and 40 vol% mHA (4.14 μm), as well as human cortical bone are also listed in this Table [44,48]. It is apparent that the hBN additions up to 20 wt% are beneficial for improving the elastic modulus of PP composites. Moreover, hBN-nHA filler hybridization further increases the modulus of composites, as expected. The PP/15%hBN-20%nHA hybrid displays a maximum modulus of 2.38 GPa, being a 69% improvement over PP. The Young’s modulus *vs*. hBN content plots for binary PP/hBN composites and ternary PP/hBN-nHA hybrids are summarized in Figure 5. From Table 2, PP exhibits a large elongation at break (>600%). The additions of 5% and 10% hBN to PP do not impair its tensile ductility. However, the elongation of PP drops to 300% by adding 15 wt% hBN and further reduces to 43% with the addition of 20% hBN. This is due to the interaction between the hBN fillers and the matrix restricts the movement of PP polymer chains at high filler loadings. This behavior is commonly observed in PP composites reinforced with fillers of micro- and nano-scale dimensions [45,49,50,51]. Hybridization of BN and nHA fillers decreases the tensile elongation of PP composites markedly, especially at high filler contents. Table 2 also reveals that conventional HDPE composites require 20 vol% mHA content to achieve a modulus of 1600 MPa and 30 vol% mHA to reach 2730 MPa. However, the modulus of PP reaches 1615 MPa by adding only 4.5 vol% hBN and further increases to 2383 MPa by adding 7.0 vol% (15 wt%) hBN and 6.67 vol% (20 wt%) nHA. The total hybrid filler content in PP/15 wt% hBN-20 wt% nHA composite is 13.67 vol%, being smaller than that of the HDPE/30 vol% mHA composite. The modulus of the PP/15 wt% hBN-20 wt% nHA hybrid is close to that of the HDPE/30 vol% mHA composite, but the tensile strength of the former is 27.5% higher than that of the latter. It is noted that the biocompatibility of the HDPE/30 vol% mHA composite is unsatisfactory; thus, filler loading of 40 vol% mHA is needed to fabricate conventional HAPEX^TM^ composite. The PP/15 wt% hBN-20 wt% nHA hybrid exhibits a higher tensile strength, but lower stiffness than the HAPEX^TM^ composite. HAPEX^TM^ can only be used for non-loading maxillofacial bone-replacements, due to its stiffness being below the modulus of load-bearing cortical bone of humans [52].

**Table 2:** Mechanical properties of PP/hBN and PP/hBN-nHA biocomposites.

Specimen	Elastic modulus, MPa	Tensile stress, MPa	Elongation at break, %
PP	1,414 ± 40	26.2 ± 0.5	>600
PP/5 wt% (2.2 vol%) hBN	1,536 ± 34	26.8 ± 0.3	>600
PP/10 wt% (4.5 vol%) hBN	1,615 ± 35	26.6 ± 0.4	>600
PP/15 wt% (7.0 vol%) hBN	1,666 ± 21	26.4 ± 0.3	300
PP/20 wt% (9.7 vol%) hBN	1,758 ± 33	26.5 ± 0.4	43.0 ± 6.3
PP/20 wt% (6.67 vol%) nHA	2,226 ± 33	30.9 ± 0.4	9.9 ± 0.6
PP/5 wt% hBN-20 wt% nHA	2,222 ± 68	27.0 ± 0.1	9.0 ± 2.0
PP/10 wt% hBN-20 wt% nHA	2,276 ± 42	28.6 ± 0.3	7.1 ± 0.6
PP/15 wt% hBN-20 wt% nHA	2,383 ± 18	26.9 ± 0.2	5.8 ± 0.3
HDPE/10 vol% mHA [44]	980 ± 20	17.3 ± 0.3	>200
HDPE/20 vol% mHA [44]	1,600 ± 20	17.8 ± 0.1	34.0 ± 9.5
HDPE/30 vol% mHA [44]	2,730 ± 10	19.5 ± 0.2	6.4 ± 0.5
HDPE/40 vol% mHA [44]	4,290 ± 17	20.7 ± 1.6	2.6 ± 0.4
Cortical bone [48]	7,000‒30,000	----------	1‒3

Table 2 reveals that the elastic modulus of the composites increases slowly by adding hBN fillers. In other words, there exists no abrupt increase in the modulus of PP composites due to hBN additions, *i.e*., no mechanical percolation. Mechanical percolation is found in the composites reinforced with fillers of very large aspect ratios, such as carbon nanotubes [53,54]. In that case, the mechanical percolation model [55,56] can be used to describe a sudden increase in the elastic or shear modulus of the composites reinforced with CNTs of very large aspect ratios. From Figure 1, the aspect ratio (width/thickness) of hBN is estimated to be ~20. Thus, hBN with a low aspect ratio cannot link with itself to form a percolative network in the PP matrix by increasing the filler content up to a critical value. Figure 3b clearly shows the absence of a percolative network in the composite with high hBN content, *i.e*., 15 wt%.

### 3.2. Cell Culture and Growth

**Figure 6:**
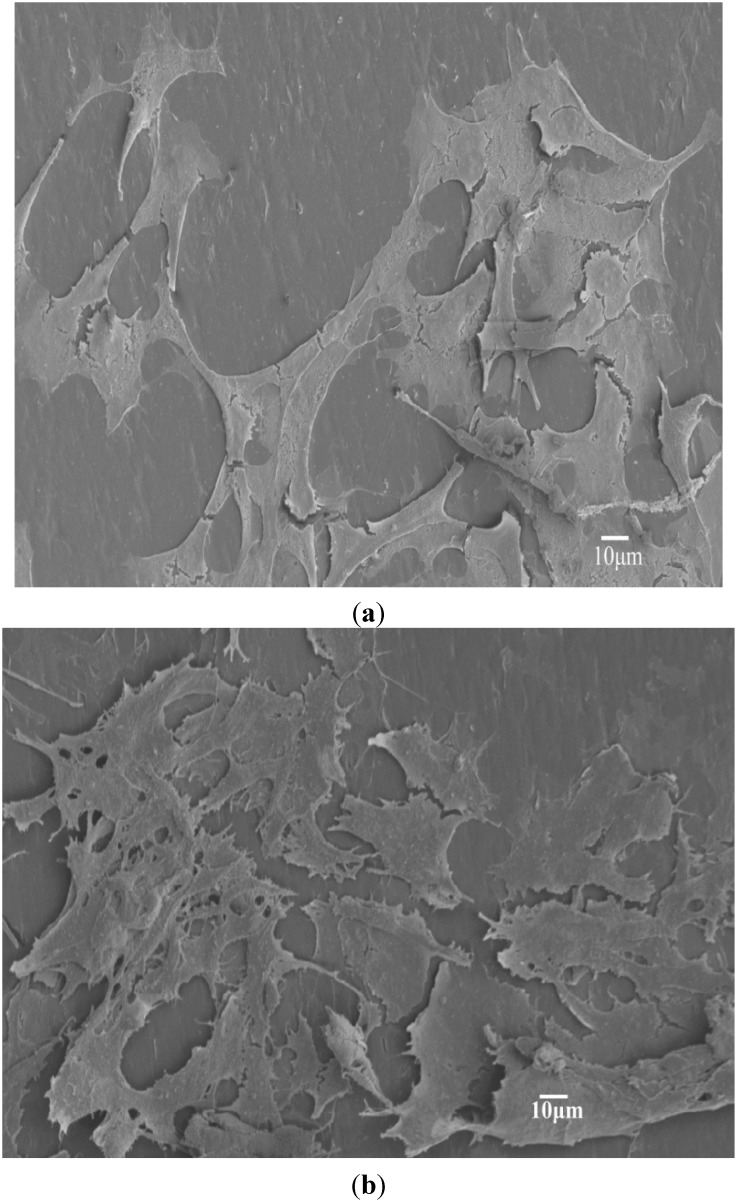
SEM images of (**a**) PP/5% hBN and (**b**) PP/10% hBN composites cultured with osteoblasts for four days showing spreading of bone cells on the composite surfaces.

**Figure 7:**
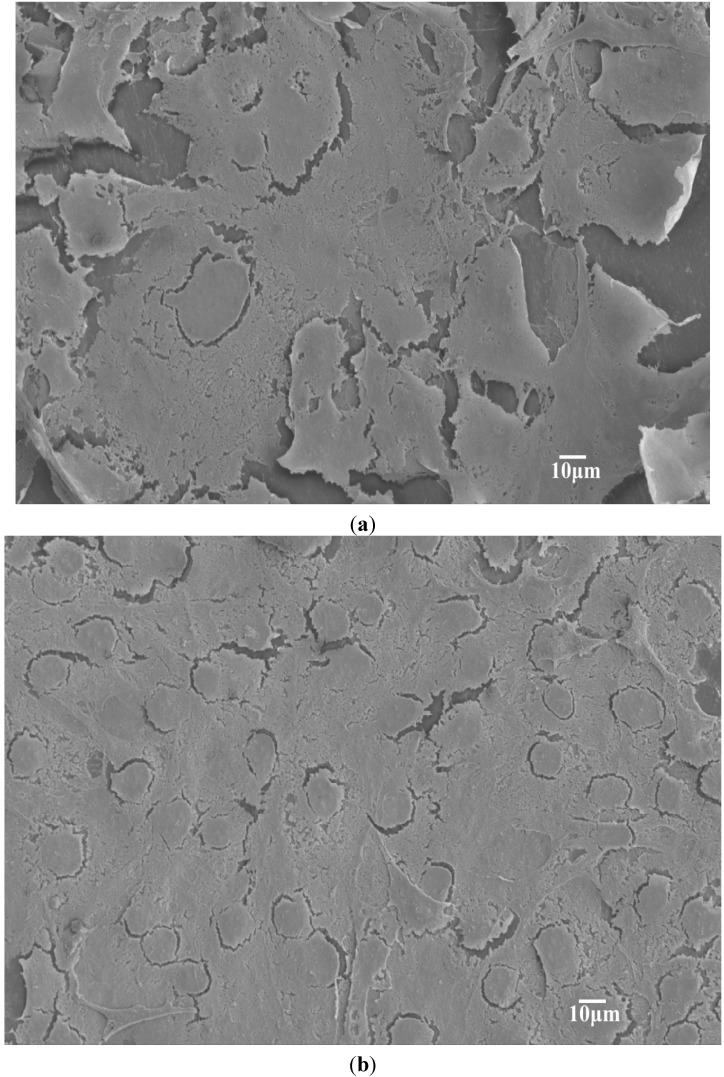
SEM micrographs of (**a**) PP/5% hBN-20% nHA and (**b**) PP/15% hBN-20%nHA hybrid composites after seeding with osteoblasts for four days. Osteoblasts almost cover entire surface of the specimens.

Figure 6a,b shows respective SEM images of the PP/5% hBN and PP/10% hBN composites after seeding with osteoblastic cells for four days. Apparently, the number of adhered cells on these samples increases with increasing hBN content. Similarly, hybrid composites also provide effective sites and support for the attachment of osteoblastic cells (Figure 7a,b). From these micrographs, the cells spread flatly on the surfaces of composite specimens. Osteoblasts anchor firmly on the sample surfaces via long filopodia. For the PP/15%hBN-20%nHA hybrid composite, cells are densely packed and piled up on each other, such that the entire composite surface is nearly covered with osteoblasts after seeding for four days (Figure 7b).

As was recognized, bone matrix is composed of nHA platelets and collagen fibers. Thus, synthetic nHA mimics the nanostructure of the inorganic phase of bone tissue and can serve as an effective seeding site for the osteoblasts. Nanohydroxyapatite with large surface areas facilitates material-bone cell interactions via protein absorption [24]. Upon adhesion to a substrate, the cell probes its environment and moves using nanometer-scale processes, such as filopodia. These interactive events lead to the formation of new bone cells. Webster *et al*. reported that synthetic nHA is biocompatible and very effective for enhancing the attachment and growth of osteoblasts. Moreover, ceramic materials, like alumina and titania, with grain sizes greater than 100 nm, have long been appreciated for their biocompatibility [57]. BN ceramic material also exhibits good biocompatibility [40], thereby promoting osteoblast adhesion.

Cell proliferation is an important health indicator for osteoblasts for ensuring the good biocompatibility of medical implants. The *in vitro* biocompatibility of binary PP/hBN composites and ternary PP/hBN-nHA hybrids was examined by the MTT assay. This assay is widely used to determine the mitochondria activity of the cells. Cytotoxicity is expressed as the percentage of cell viability by using the following relation:

Cell viability (%) = 100 [OD of sample cells/OD of control]
(1)

**Figure 8:**
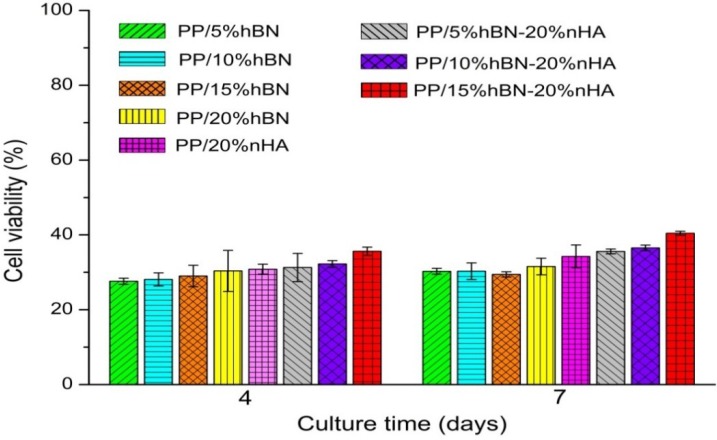
Cell viability of osteoblasts grown on PP/20% nHA, PP/hBN and PP/hBN-nHA composites after seeding for four and seven days. PP/15%hBN-20%nHA hybrid exhibits the highest viability.

The cellular viability of composite specimens is shown in Figure 8. It can be seen that cellular viability for the PP/hBN and PP/hBN-nHA composites increases with cell culture time from four to seven days. These specimens show low viability values. From the literature, the MTT test tends to give lower cellular viability due to formazan crystals clumping together with BN tubes and the water-insoluble nature of MTT-formazan [37,39]. However, (2-(4-iodopheneyl)-3-(4-nitophenyl)-5-(2,4-disulfophenyl)-2H-tetrazolium monosodium salt (WST-1) assay can yield higher cellular viability because of the water-soluble nature of its formazan product. It is likely that the interference of hBN with MTT-formazan also causes lower cellular viability of the PP/hBN and PP/hBN-nHA composites (28%–30%). Finally, the PP/hBN-nHA hybrid composite system exhibits a slightly higher proliferation rate than binary PP/hBN composites for all testing time intervals. This derives from the synergistic effect of the individual components of hybrid fillers. The results imply that all composite materials studied have no toxicity for the adhesion and growth of osteoblastic cells. From the tensile, cell culture and proliferation tests, it can be concluded that the PP/15%hBN-20% nHA hybrid has great potential for application in maxillofacial surgery.

## 4. Conclusions

This article presented the design and testing of binary and hybrid composites for human bone replacements. Binary PP/hBN and ternary PP/hBN-20%nHA composites were successfully prepared by melt mixing and injection molding techniques. Hybrid nanocomposites inherited the property of individual fillers by producing materials with better biocompatibility and mechanical properties. The results showed that the elastic modulus of PP composites increases with increasing the hBN content. Hybridization of hBN with nHA further enhances the elastic modulus of PP composites. The hBN and/or nHA additions reduce tensile ductility of the PP biocomposites. Finally, cell cultivation and MTT assay results revealed that the osteoblasts can attach and proliferate on binary PP/BN and ternary PP/BN-20%nHA composites.
